# ATP-Dependent Chromatin Remodeling Factors and Their Roles in Affecting Nucleosome Fiber Composition

**DOI:** 10.3390/ijms12106544

**Published:** 2011-10-06

**Authors:** Paolo Piatti, Anette Zeilner, Alexandra Lusser

**Affiliations:** Division of Molecular Biology, Innsbruck Medical University, Biocenter, Fritz-Pregl Strasse 3, 6020 Innsbruck, Austria; E-Mails: paolo.piatti@i-med.ac.at (P.P.); anette.zeilner@i-med.ac.at (A.Z.)

**Keywords:** chromatin, histone variant, chromatin remodeling factor, centromere, linker histone, chromatin assembly

## Abstract

ATP-dependent chromatin remodeling factors of the SNF2 family are key components of the cellular machineries that shape and regulate chromatin structure and function. Members of this group of proteins have broad and heterogeneous functions ranging from controlling gene activity, facilitating DNA damage repair, promoting homologous recombination to maintaining genomic stability. Several chromatin remodeling factors are critical components of nucleosome assembly processes, and recent reports have identified specific functions of distinct chromatin remodeling factors in the assembly of variant histones into chromatin. In this review we will discuss the specific roles of ATP-dependent chromatin remodeling factors in determining nucleosome composition and, thus, chromatin fiber properties.

## 1. Introduction

Chromatin is an extremely complex structure that serves to compact eukaryotic DNA in order to comply with the size restrictions of the nucleus. In addition, the way in which chromatin is organized and in which its arrangement is modulated endows it with an extraordinary regulatory potential. At its most basic level of organization, chromatin consists of repeating spherical particles termed nucleosomes. Nucleosomes are formed by the wrapping of 147 bp of DNA in 1.7 left-handed superhelical turns around a core of small, evolutionary conserved, highly basic histone proteins [1]. Two molecules each of the histones H3 and H4 interact via the so-called “histone-fold” domains to generate a protein tetramer, which associates with two heterodimers of the histones H2A and H2B to form the nucleosome core [1]. Nucleosomes are connected by short stretches of linker DNA resulting in a fiber with a diameter of ~10 nm that has a beads-on-a-string-like appearance [2,3]. Although this structure may seem uniform from a superficial perspective, a tremendous amount of research during the past decades has provided ample evidence that nucleosomes can differ from each other with respect to their structure, the type of histones that they contain as well as the nature and extent of chemical modifications on both the DNA and histones. In addition, the positioning of the nucleosomes along the DNA can show striking variation, including regular arrangements with constant spacing (e.g., in constitutive heterochromatin), irregular arrays of nucleosomes (typically in active genes) or regions that are devoid or depleted of nucleosomes (e.g., at enhancers and promoters) [4,5]. Importantly, chromatin structure is not static. On the contrary, the organization and composition of chromatin is constantly changing thereby facilitating or preventing access for DNA-utilizing proteins to their substrate. In this review we will discuss some of the mechanisms that contribute to the shaping of chromatin structure not only at the level of the 10 nm fiber but also in higher-order levels of chromatin organization. We will give special attention to the ATP-dependent chromatin remodeling machines and their diverse roles in modulating the composition of nucleosomes and chromatin fibers.

## 2. Chromatin Remodeling Machines and Their Impact on Nucleosome Structure

Chromatin organization is regulated on various levels and by a multitude of diverse proteins and non-coding RNAs. On one hand, enzyme complexes that use DNA for transcription, replication, recombination or repair actively contribute to changing chromatin structure. For instance, RNA and DNA polymerases travel along the DNA double helix and by doing so introduce torsional stress that can promote the loss of histones ahead of them and facilitate the reassembly of nucleosomes in their wake [6]. Although most of this stress is constantly released by the action of topoisomerases, it is likely that DNA-utilizing processes exert distinct effects on local as well as regional chromatin structure. Other mechanisms that profoundly affect chromatin structure are posttranslational modifications of nucleosomal histones, the incorporation of so-called variant histone proteins and of other non-histone architectural proteins, such as high mobility group (HMG) proteins, as well as the energy-consuming remodeling of nucleosomes by ATP-dependent remodeling machines [7–10]. ATP-dependent chromatin remodeling factors typically are large protein complexes that contain an ATPase subunit, which belongs to the sucrose non-fermenting 2 (SNF2) family of ATPases/helicases [11,12]. SNF2-like ATPases can be grouped into 23 subclasses according to sequence differences in their ATPase domains and the presence of additional protein motifs [11]. The best-studied chromatin remodeling factors belong to the SWI/SNF (switch/sucrose non-fermenting), the ISWI (imitation switch), the CHD (chromo helicase DNA binding) and the INO80 (inositol auxotroph 80) subfamilies [10,13–17].

### 2.1. The Role of ATP-Dependent Chromatin Remodeling Factors in Nucleosome Positioning

Several recent studies that mapped the positions of nucleosomes at a genome-wide level in different organisms and cell types have reported the existence of rather well conserved patterns of nucleosome occupancy in particular at the 5′ and 3′ ends of genes (e.g., [18–22]). Using micrococcal digestion combined with deep-sequencing technology, it was shown for yeast, *Drosophila* and humans that promoters are commonly marked by a nucleosome-free or depleted region (NDR) upstream of the transcriptional start site (TSS). Furthermore, the first nucleosome downstream of the TSS (+1 nucleosome) usually occupies a distinct position, which is ~50 bp downstream of the TSS in yeast and at ~ +135 bp in *Drosophila* and humans [4,5]. Another NDR appears to be distinctive of 3′-ends of genes. Upstream of this NDR a positioned nucleosome is usually detected although the latter appears not to be universally conserved [4,5,23]. Although DNA sequence is likely to influence some of the nucleosome positions, in particular the NDRs, it was postulated that ATP-dependent chromatin remodeling machines play an important role in determining nucleosome positions *in vivo* [4,5,24]. This is especially likely for nucleosomes that occupy energetically unfavorable positions.

Chromatin remodeling enzymes are well equipped to carry out this task. In many elegant *in vitro* studies, it has been demonstrated that by using the energy derived from hydrolyzing ATP, these enzymes can break and/or establish histone-DNA contacts. The results of these actions are manifold and dependent on the type of remodeler as well as on the functional context [10,25–27]. Numerous studies exploring the effects of deletion or knock-down of chromatin remodelers have found wide-spread gene regulation defects [28]. These effects can at least in part be attributed to a role of these factors in positioning and remodeling of nucleosomes. Two SNF2 subfamilies in particular, the ISWI and the CHD families, have been shown to be able to move nucleosomes to different translational positions along the DNA (“sliding”) [29–34]. Consistent with this function, ISWI and CHD type enzymes have been shown to be associated with active genes [35–38]. They have roles in remodeling nucleosomes in the vicinity of the TSS [37–40], but they seem also involved in regulating nucleosome positioning at the 3′-end of genes. In yeast it was observed that loss of Isw2 resulted in increased production of non-coding transcripts. These transcripts originated from mis-oriented transcription as a result of aberrant nucleosome positioning at the 3′-end of Isw2 target genes [37]. Likewise, yeast Chd1 was shown to be involved in organizing the nucleosomal fiber at the 3′-end of genes, since deletion of CHD1 resulted in transcription termination defects and aberrant nucleosomal arrangements at the 3′-ends of the CYC1 and ASC1 genes [41]. Very recently, the Mi-2/CHD3-related ATPase Mit1 (Mi2-like protein interacting with Clr three 1), which is part of the SHREC (Snf2/Hdac-containing Repressor Complex) complex in *Schizosaccharomyces pombe*was shown to profoundly affect nucleosome positioning globally and at specific heterochromatic sites [23,42].

Chromatin remodeling complexes of the SWI/SNF family have also been extensively characterized *in vitro* and *in vivo*. One salient feature of this type of remodeler is its ability to disrupt nucleosome structure more profoundly than ISWI and CHD enzymes (e.g., [43–46]). SWI/SNF enzymes can eject histones from nucleosomes, they can transfer dimers and tetramers to other DNA molecules (e.g., [43,47–49]) and they can catalyze nucleosome sliding reactions [10,50]. Thus, *in vivo* SWI/SNF ATPases have been identified as crucial regulators of gene activation, and they have been shown to be able to generate NDRs [51].

Almost all SNF2-type motors are part of (large) protein complexes. The accessory subunits can gravely impact on the biochemical properties of a remodeler complex. For instance, association of the ISWI motor protein with the ATP-dependent chromatin assembly factor 1 (Acf1) subunit, strongly stimulates the efficiency by which it can assemble and remodel nucleosomes [52]. In a similar manner the chromatin remodeling activity of the SWI/SNF ATPases BRG1 (brahma related gene 1) and hBRM (human brahma) are significantly enhanced by the INI1 (integrase interactor 1) and the brahma-associated factors BAF155 and BAF170 complex subunits [53]. Nevertheless, a recent study demonstrated that the ATPases themselves exhibit strikingly different characteristics with respect to their nucleosome sliding properties. When *Drosophila* ISWI and CHD1 as well as human Snf2H, Brg1 and Mi-2 (dermatomyositis specific autoantigen Mi-2) were tested side by side in an *in vitro* sliding assay, each remodeler moved the nucleosome to different positions although the underlying DNA sequence was the same in all cases [34]. Hence, it is conceivable that *in vivo* different chromatin remodeling factors may establish specific local nucleosome positions in addition to histone displacement. The action of these enzymes, therefore, will not only facilitate but also impede the access of factors to their binding sites on the DNA.

### 2.2. Chromatin Remodeling Factorsin Replication-Coupled Nucleosome Assembly

During S-phase, when the DNA is replicated, chromatin is completely disassembled and nucleosomes are reformed at the nascent daughter strands. Thereby, newly synthesized histones must be incorporated to complement the “old” histones that are reused in the newly established nucleosomes [54,55]. ATP-dependent factors are likely to adopt a critical position within the DNA replication process. They are known to not only slide and restructure existing nucleosomes but also to mediate the formation of new nucleosomes or change the histone composition of nucleosomes [8,56]. ISWI-containing remodeling complexes, such as ACF (ATP-dependent chromatin assembly and remodeling factor)and RSF (remodeling and spacing factor), and CHD1 have been demonstrated to be able to generate nucleosome arrays *in vitro* from purified histones and DNA. ACF and CHD1 perform this reaction in conjunction with the histone chaperone NAP-1 (nucleosome assembly protein 1), while RSF does not require a chaperone [52,57–60].

Despite the well-characterized biochemical activities of chromatin remodeling factors it is rather surprising that information about their involvement in replication-coupled chromatin assembly *in vivo* is still limited. To date, only ISWI-type enzymes have been linked to nucleosome formation during S-phase. In *Drosophila* the inactivation of the ACF complex by deletion of its Acf1 subunit resulted in an acceleration of S-phase caused by a shortening of heterochromatin replication timing [61]. Similarly, the human ISWI homolog SNF2h was proposed to play a role in replication-coupled heterochromatin assembly [62–64]. In this case, two different SNF2h-containing complexes appear to be important, since knock-down of the ACF1 subunit of the human ACF complex inhibited progression through S-phase [63], while a complex containing Snf2h and the Williams syndrome transcription factor (WSTF) targeted SNF2h to heterochromatin by interaction with proliferating cell nuclear antigen (PCNA), which is a processivity factor of DNA polymerase [62,64]. Thus, ISWI enzymes appear to be involved in replication-coupled heterochromatin assembly. However, in light of more recent studies implicating ISWI in the incorporation of the linker histone H1 (see below), the above-mentioned observations might not fully support this conclusion. A recent report identified the mammalian SNF2-type ATPase SMARCAD1 as an important regulator of global DNA replication-associated histone deacetylation. As a consequence of SMARCAD1 knock-down, heterochromatin establishment, in particular histone H3 lysine 9 trimethylation and HP1 binding was perturbed [65]. Thus, while SMARCAD1 appears to play a crucial role in thedeacetylation of newly incorporated histones, which are acetylated, it seems not to be directly involved in histone deposition. Therefore, to date no chromatin remodeler has been unequivocally demonstrated to mediate the reassembly of either heterochromatin or euchromatin in the course of DNA replication *in vivo.*

### 2.3. Incorporation of Linker Histone H1

The linker histone H1 associates with DNA at nucleosome entry/exit sites and thereby affects the folding of the 10 nm nucleosomal fiber into higher-order structures with a diameter of about 30 nm [66,67]. It is assumed that the 30 nm fiber makes chromatin less accessible to DNA binding factors and is thus largely refractory for processes such as transcription. Although several recent studies have made considerable progress in elucidating the structure of *in vitro* reconstituted 30 nm fibers [68–71], their *in vivo* organization appears to be heterogeneous and is still poorly understood [72,73]. This may be due in part to the highly dynamic behavior of H1 *in vivo*. While the core histones H3 and H4 typically remain bound to the chromatin over several cell generations, H1 turn-over occurs within seconds [74–77].

Several lines of evidence point to a critical role for ATP-dependent chromatin remodelers in H1 assembly. First, it was shown that *in vitro* ACF and ISWI but not the CHD-type factor CHD1 can generate periodic H1-containing nucleosome arrays [58,78,79]. Second, in *Drosophila*, deletion of ISWI resulted in global decondensation of the transcriptionally hyperactive single X chromosome in salivary glands of male larvae, and overexpression of a dominant negative allele of ISWI led to striking alterations in the appearance of autosomes as well as sex chromosomes. These changes were accompanied by a decrease in chromosomal H1 levels [80,81]. These findings suggest that ISWI is required for the incorporation of H1 into chromatin *in vivo*. ISWI is part of multiple chromatin remodeling complexes, and in a study of the largest subunit of the ISWI-containing NURF (nucleosome remodeling factor) complex, Nurf301, it was shown that *Nurf301* mutant alleles resulted in a decondensation phenotype of the male X chromosome similar to that of an *Iswi* mutation [82,83] suggesting that NURF might be involved in H1 incorporation. However, *Nurf301* mutation also causes the upregulation of roX RNA, which is a central component of the male specific lethal (MSL) complex, which is required for dosis compensation in *Drosophila* males. Mutations of roX suppressed the puffing phenotype of the *Nurf301* mutants [83], and it is not clear at this point whether the derepression of roX in *Nurf301* mutants and/or H1 incorporation defects are responsible for the distortions in chromatin structure observed in the absence of functional Nurf301. There is also evidence that another ISWI-containing complex, ACF, might contribute to H1 incorporation. In mutants for the signature subunit of ACF, Acf1, a global shortening of nucleosomal repeat length was observed [61]. Such changes also occur when H1 levels are strongly reduced [77,84,85] and therefore might argue for an involvement of ACF in H1 assembly. Given that *Acf1* mutants do not exhibit structural defects on the male X chromosome, it is possible that H1 loading is achieved by the combined actions of different ISWI complexes. Regardless of the type of complex, these studies provide an example that ATP-dependent chromatin remodeling not only affects the structure of the basic nucleosome fiber but also has important functions in modulating higher-order chromatin folding.

### 2.4. Incorporation of Variant Histones

**Figure 1:**
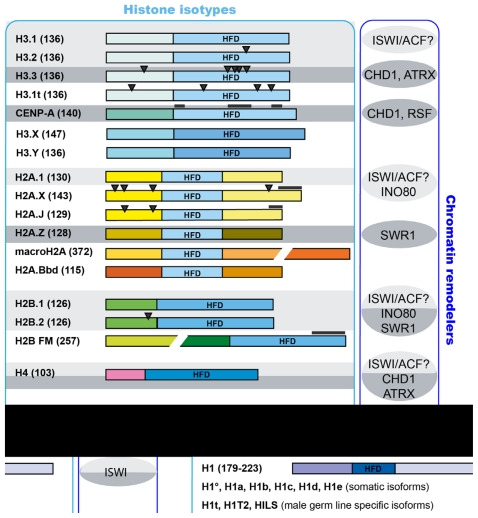
Schematic representation of mammalian histone isotypes (left panel) and of the respective remodeling enzymes that have been linked to their incorporation (right panel). Numbers in parenthesis represent the amino acid sequence lengths of the histone proteins. Identical colors indicate identical amino acid sequences. Replication-coupled histone incorporation is denoted by light grey shading, replication-independent assembly is indicated by dark grey shading [91–93].

A major manifestation of chromatin dynamics is the constant turn-over of chromosomal histones. Even in post-mitotic cells histones are continually exchanged. During replication-coupled assembly the so-called “canonical” histones are incorporated. These histones are encoded by multiple gene copies in higher eukaryotes, and their expression is tightly controlled to reach its maximum in S-phase [86]. Canonical histones are not incorporated by replication-independent mechanisms. To this end, variant histones are used [87]. Consequently, in post-mitotic cells canonical histones are gradually replaced with histone variants. For instance, measurements in long-lived neurons have shown that ~80% of all H3 histones are of the H3.3 variant type [88]. An important process that requires the replication-independent assembly of histones is transcription. It has been shown that fast and profound histone loss occurs at highly transcribed genes, such as the heat shock protein 70 (Hsp70) genes in *Drosophila* [39]. Moreover, measurements of the incorporation of GFP-tagged histone H3.3 have revealed that H3.3 accumulates at transcriptionally active sites [89,90]. Some histone variants are highly similar in sequence to their replication-coupled counterparts. For instance, H3.3 differs from H3.2 with only four amino acids. On the other hand, there are histone isotypes, such as macroH2A or H2A.Bbd (H2A Barr body deficient), whose sequence deviates considerably from the canonical histone type (Figure 1) [91–93].

Multiple studies have shown that assembly and exchange of histones require the concerted action of histone chaperones and ATP-dependent chromatin remodeling factors in the context of both replication-coupled as well as replication-independent processes [56,94]. It has also become apparent in recent years that *in vivo* the incorporation of individual histone variants requires distinct types of ATP-dependent factors together with specialized histone chaperones. Although the mechanisms of incorporation of a number of variants still await discovery, considerable progress has been made in elucidating the critical factors involved in the incorporation of variant histones, such as H3.3, the centromere-specific H3 variant CenH3 and the H2A variant H2A.Z [8,92,95,96].

Regardless of the mechanisms of incorporation, histone variant composition of chromatin correlates with its functional properties and affects chromatin dynamics locally or in a global manner [92]. For instance, H3.3 and the H2A variant H2A.Bbd colocalize predominantly with transcriptionally active chromatin, CenH3 is only found at centromeres, where it generates a chromatin structure suitable to the formation of the kinetochore, and the variant histone macroH2A is distinctive of the transcriptionally silent X-chromosome of female mammals [92]. Different histone isotypes may affect nucleosome and chromatin structure and dynamics in various ways. They may be subject to distinct posttranslational modifications [97], alter interactions with components of the DNA-utilizing machineries, or they may affect the structural properties of variant-containing nucleosomes in a way that makes the underlying DNA sequence more or less permissible for a certain functional state.

### 2.5. Chromatin Remodelers and H3.3

A recent crystal structure analysis revealed that incorporation of H3.3 into nucleosomes instead of the replication-coupled H3.1 and H3.2 forms does not lead to obvious structural effects on the nucleosome [98,99]. Nevertheless, nucleosomes purified from chicken erythroid cells were found to be less stable when containing H3.3 [99]. Therefore, it was proposed that a destabilization of nucleosomes by H3.3 may promote the accessibility of active genes and regulatory regions [99]. Consistent with this idea is the observation that increased levels of H3.3 are detected over active genes and at transcription factor binding sites [100–102]. Despite its broad distribution in nuclear chromatin, H3.3 is not essential for viability of *Drosophila* [103,104]. Yet it is required for germ cell development, and male and female flies with mutated H3.3 are sterile [103,104].

**Figure 2:**
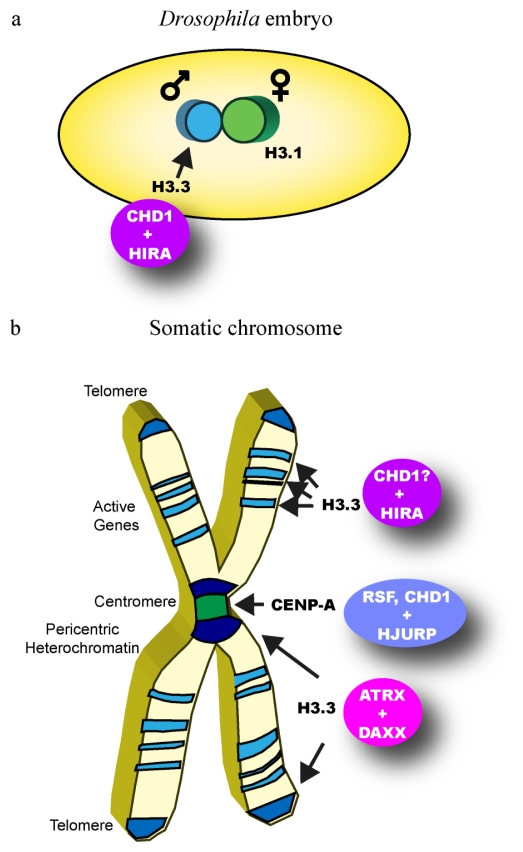
Replication-independent assembly of histone H3 variants. (**a**) The chromatin remodeler CHD1 cooperates with the H3.3-specific histone chaperone HIRA to incorporate H3.3 into the paternal pronucleus at fertilization in *Drosophila* embryos. The maternal pronucleus does not require chromatin reorganization and contains predominantly H3.1. (**b**) Different chromatin remodeling complexes in conjunction with specific histone chaperones incorporate H3.3 and CENP-A at distinct chromosomal sites. Dark blue shading indicates H3.3 incorporation into telomeric and pericentric heterochromatin, respectively. Lighter blue shading indicates H3.3 assembly at genic locations. Green shading denotes CENP-A incorporation into chromatin at the centromere.

H3.3 is predominantly incorporated co-transcriptionally with the exception of an early instance in development. At fertilization the chromatin of the paternal pronucleus requires drastic reorganization. In this situation, global reassembly of nucleosomes must occur in order to replace sperm-specific DNA packaging proteins, the protamines, that are responsible for organizing sperm chromatin. It has been shown in *Drosophila* and mouse embryos that during paternal pronuclear rearrangement the histone variant H3.3 but not H3.1 is loaded onto the DNA [105,106]. In *Drosophila*, the protamine/histone exchange takes place prior to the onset of DNA replication and transcription and thus, H3.3 deposition must be independent of a transcription-linked process. The histone chaperone HIRA (histone cell cycle regulation defective homolog A) was identified as a crucial factor for the loading of H3.3 in this process, since mutation of HIRA abrogated the incorporation of H3.3 into the paternal chromatin [105,107]. Similar but not identical defects were observed when the ATP-dependent chromatin remodeler CHD1 was deleted in the fly. The absence of CHD1 resulted in the accumulation of H3.3 at the nuclear periphery of paternal pronuclei indicating that CHD1 is required for correct deposition of H3.3 [108]. Thus, CHD1 and HIRA appear to work together in the transcription-independent incorporation of H3.3 at this specific developmental instance, a notion that is corroborated by the observation that both factors physically interact in early *Drosophila* embryos [108] (Figure 2a).

CHD1 may also have a role in the transcription-dependent incorporation of H3.3. This idea is supported by the finding that in *Chd1*-defective *Drosophila* embryos, aberrant H3.3 localization is detected in transcriptionally active syncytial nuclei [108]. In addition, knock-down of CHD1 in mouse embryonic stem (ES) cells resulted in compromised pluripotency probably due to an observed decrease of euchromatin and a concomitant spreading of heterochromatin [109]. Although H3.3 incorporation was not tested in this study, the fact that H3.3 normally is enriched in euchromatin may point to a defect in generating proper H3.3-containing nucleosomes. Interestingly, whole-genome H3.3 mapping experiments have revealed that mammalian HIRA is necessary for H3.3 enrichment at active and repressed genes [100,110,111] (Figure 2b).

A number of recent reports have implicated another SNF2-type chromatin remodeling factor, the *α*-thalassemia/mental retardation syndrome X-linked (ATRX) protein, in H3.3 incorporation into chromatin. ATRX was shown to be required for loading of H3.3 into chromatin at telomeres in mouse ES cells [100,110,111] and at pericentric heterochromatin in mouse embryonic fibroblasts [112] (Figure 2b). Thorough biochemical analyses revealed that ATRX cooperates with a novel H3.3-specific histone chaperone termed DAXX (death domain associated protein) [100,111,112]. Thus, to date two different ATP-dependent chromatin remodeling factors have been implicated in H3.3 incorporation. Both factors function together with distinct chaperones (CHD1 with HIRA, ATRX with DAXX) to generate H3.3-containing nucleosomes in specific nuclear neighborhoods or developmental occasions reflecting a highly complex assembly machinery that enables the formation of functionally distinct chromatin areas (Figure 2b).

### 2.6. Chromatin Remodelers and the Assembly of Centromeric Chromatin

The histone H3 variant CenH3 (also known as CENP-A, CID, Cnp1, Cse4) is incorporated into chromatin at the centromeres in a transcription-independent fashion [113,114]. Its presence at the centromere is thought to identify the region for kinetochore assembly, since centromeric DNA sequences are not conserved between organisms and therefore not likely to contribute to this task [113,115]. The assembly of centromeric chromatin appears to involve a great number of proteins. Despite considerable research efforts over the past years it is still not entirely clear as to which factors are directly involved in CenH3 assembly and which ones act in an indirect manner [113–116]. For example, a recent study showed that the yeast SWI/SNF complex acts to remove the yeast centromeric histone Cse4 from nucleosomes outside of the centromere. Thus, it acts to confine Cse4 to the single centromeric nucleosome that defines centromeres in *Saccharomyces* but is not involved in the loading of Cse4 [117]. A large step towards elucidating centromeric chromatin assembly has been made with the discovery of a CenH3-specific histone chaperone, termed HJURP (Holliday junction recognition protein) [118,119]. HJURP has been demonstrated to directly interact with soluble CenH3. Moreover, knock-down of HJURP led to the loss of CenH3 signals at the centromere [118,119]. Interestingly, HJURP is distantly related to the *Saccharomyces cerevisiae* Scm3 protein, which also has been shown to act as a CenH3 chaperone [120,121], but does not have any apparent homologs in *Drosophila* [122].

Among the ATP-dependent factors the ISWI-containing complex RSF was reported to interact with CenH3-containing mononucleosomes in human cells and to play a role in the incorporation of human CenH3 [123]. However, as the effects of RSF knock-down are relatively mild, it is likely that there are additional proteins involved [123]. Indeed, in chicken DT40 cells as well as in fission yeast, CHD1 and its homolog Hrp1, respectively, have been linked to a role in the assembly of CenH3-containing nucleosomes [124,125]. In contrast, no such role could be demonstrated for CHD1 in *Drosophila* [126]. Similarly, no centromere defects have been reported for *Drosophila* Rsf1 mutants [127]). Thus, flies appear to neither possess a *bona fide* HJURP homolog, nor are the roles of CHD1 and RSF in CenH3 incorporation conserved. Instead, a *Drosophila*-specific factor, termed CAL1 (chromosome alignment defect 1), was demonstrated to interact with CenH3 and to be required for its loading to chromatin [128]. To date, no ATP-dependent factor was found to participate in this process. These results indicate that different organisms might use different mechanisms and factors to ensure CenH3 assembly at centromeres.

### 2.7. Chromatin Remodelers and H2A.Z Exchange

The replacement of H2A/H2B dimers in nucleosomes with dimers containing the variant histone H2A.Z/H2B is a common event in all eukaryotes. Its importance is emphasized by the fact that H2A.Z is essential for viability in *Drosophila*, *Tetrahymena* and mouse [129–131]. Incorporation of H2A.Z into nucleosomes does not result in large structural alterations, but nevertheless causes some intriguing changes. On one hand, H2A.Z-containing nucleosomes possess a larger acidic patch at the surface of the octamer that was proposed to serve in the interaction with the H4 N-terminal tail of a neighboring nucleosome [132]. Indeed, *in vitro* H2A.Z-containing nucleosome arrays were shown to be more tightly compacted than H2A-containing nucleosomes [133]. These observations are consistent with the results from genome-wide analyses of H2A.Z distribution that found that H2A.Z is present in heterochromatic areas of the genome [134,135].

On the other hand, however, the interface between H2A.Z/H2B and H3/H4 dimers in the crystal structure was found to be slightly less stable than in H2A-nucleosomes and thus may render these nucleosomes more prone to disruption [132]. Aside from heterochromatic sites, H2A.Z is particularly enriched in nucleosomes at the transcription start sites of genes [99,136]. In line with the predictions of the crystal structure analysis, H2A.Z-containing nucleosomes isolated from chicken erythroid cells displayed reduced stability [99,136]. However, H2A.Z-containing nucleosomes were only less stable when they simultaneously contained H3.3 but not when they contained H3 [99,136]. Hence, it appears that the combination of H2A.Z with either H3.3 or H3.1 confers quite distinct properties to these nucleosomes. This may in part explain the seemingly contradictory presence of H2A.Z in heterochromatin and euchromatin.

H2A.Z replacement is carried out by a dedicated ATP-dependent remodeler, termed SWR1 [137–141] (Figure 1). SWR1 belongs to the INO80 subclass of chromatin remodelers and is characterized by a split ATPase domain [15]. It is part of a multiprotein complex, which also contains subunits necessary for H2A.Z recognition and for binding to acetylated H3/H4 [142]. A recently published study demonstrated that the second member of the INO80 subfamily, INO80, also affects H2A.Z-containing nucleosomes [143] (Figure 1). Interestingly, INO80 performs the opposite reaction to SWR1 by catalyzing the exchange of H2A.Z/H2B dimers for H2A/H2B. Deletion of INO80 in yeast resulted in aberrant localization of H2A.Z in promoter and coding regions. Moreover, replication fork progression defects of *δino80* mutants were alleviated by reduced expression of H2A.Z, suggesting that the misincorporation of H2A.Z in the absence of INO80 causes the observed defect [143].

A number of histone chaperones have been implicated in H2A.Z dynamics. Nap1 was shown to enable H2A.Z/H2B dimer exchange in an *in vitro* reaction [144] and was also detected in purified SWR1 complex fractions [137]. In *S. cerevisiae*, another H2A.Z-specific chaperone, termed Chz1, was identified, which together with Nap1 represents the two major H2A.Z/H2B chaperones in this organism [145]. Interestingly in the absence of both Chz1 and Nap1 additional proteins, such as the FACT complex, the karyopherin Kap114 and two peptidylprolyl *cis-trans* isomerases termed Fpr3 and Fpr4, were shown to interact with H2A.Z/H2B dimers [145]. Further studies in yeast indicated that NAP-1 is important for chaperoning the soluble pool of H2A.Z, whereas Chz1 does not interact with H2A.Z in the cytoplasm [146].

## 3. Do Chromatin Remodeling Factors Incorporate Non-Histone Chromosomal Proteins?

As discussed above, ATP-dependent remodeling factors are crucial components of the machineries that deposit histones and generate patterns of nucleosomes with diverse composition (Figure 1). Apart from histones and their variants, however, there are other abundant non-histone architectural proteins, such as HMG proteins or heterochromatin protein 1 (HP1), that associate with the chromatin and shape its structure and dynamics. Do these factors also require motor proteins to bind correctly to the nucleosome fiber? Although this intriguing question has not been investigated in great detail so far, some recent studies provide evidence that suggests this may indeed be the case.

The HMG proteins are among those non-histone architectural proteins that have been studied most extensively [9,147,148]. HMG proteins generally act to decrease the compactness of the chromatin fiber and therefore render chromatin more accessible to regulatory factors [147,149]. They bind to chromatin in a highly dynamic and reversible way either by directly contacting the nucleosome and/or via co-factors. There are three subfamilies of HMG proteins, termed HMGA, HMGB and HMGN [9,148]. Members of the HMGN group, in particular, have been shown to bind to nucleosomes at the entry/exit sites of the DNA and therefore compete with the linker histone H1 for nucleosome binding sites [150,151]. They also exhibit exchange dynamics that are similar to those of H1 [152]. As detailed above, H1 incorporation into chromatin is strongly dependent on the ISWI chromatin assembly factor [61,81]. By analogy, HMGN proteins might also require an ATP-dependent factor for efficient chromatin association. In a recent study addressing the effects of HMGN1 and HMGN2 on chromatin remodeling by the ATP-dependent factors ACF and the SWI/SNF-family protein BRG1, it was shown that ACF can assemble extended periodic nucleosome arrays containing HMGN proteins *in vitro* [153]. Although *in vivo* studies have not yet been carried out, these experiments provide an intriguing hint for a possible function of ACF and potentially other chromatin remodeling factors in the assembly of not only histones but also of non-histone architectural proteins into chromatin.

Another candidate remodeling factor for the incorporation of non-histone chromosomal proteins may be ATRX. As discussed above, ATRX has recently been characterized to be required for the incorporation of histone H3.3 into pericentric and telomeric chromatin [100,110–112]. Yet, ATRX has also been shown to physically interact with HP1, which is an abundant protein localized in heterochromatin [154–157]. Two recent studies provided evidence for a function of ATRX in the loading of HP1 to chromatin. In *Drosophila*, deletion of ATRX resulted in the loss of HP1α from pericentricβ-heterochromatin [157]. Along the same lines, depletion of ATRX in mouse ES cells led to a strong decrease of HP1α localization at telomeric chromatin [110]. Although these findings point to a role of ATRX in the association of HP1α with heterochromatin, biochemical studies will be necessary to determine, if indeed ATRX uses its catalytic activity to incorporate HP1 or if the observed phenotypes are the result of recruitment defects. Previous *in vitro* experiments have demonstrated that the ACF remodeling factor greatly stimulates the association of HP1 with reconstituted nucleosome arrays. This stimulation, however, was found to be dependent on the Acf1 subunit of the complex and did not involve ATP-hydrolysis [158]. Nevertheless, although the evidence is somewhat circumstantial at the moment, it will be interesting to elucidate whether non-histone architectural proteins are actual targets of ATP-dependent chromatin remodeling machines.

## 4. Conclusion

Nucleosome assembly is not only necessary for preserving chromatin structure and, thus, genome integrity, it is also a process that strongly impacts on the functional properties of the nucleosomal fiber. In particular, the incorporation of specific histone variants into nucleosomes on one hand serves to modulate the biophysical properties of nucleosomes but can also endow nucleosomes with distinct abilities to interact with regulatory factors or to receive specific posttranslational modification marks. Moreover, the presence of histone variants at functionally distinct regions in the genome has been postulated to serve as a means to transmit epigenetic information across cell generations [159,160]. With the discovery of several novel histone incorporation pathways over the past years, it has become clear that ATP-dependent chromatin remodeling factors in conjunction with specific histone chaperones act at the center of these processes. It will be interesting to see in the future, whether dedicated partnerships of ATP-dependent motor proteins with histone chaperones exist for the assembly of all histone variants and even non-histone architectural proteins and what the biological consequences of their actions are.
